# Gene Knock-Outs of Inositol 1,4,5-Trisphosphate Receptors Types 1 and 2 Result in Perturbation of Cardiogenesis

**DOI:** 10.1371/journal.pone.0012500

**Published:** 2010-09-01

**Authors:** Keiko Uchida, Megumi Aramaki, Maki Nakazawa, Chihiro Yamagishi, Shinji Makino, Keiichi Fukuda, Takeshi Nakamura, Takao Takahashi, Katsuhiko Mikoshiba, Hiroyuki Yamagishi

**Affiliations:** 1 Department of Pediatrics, Keio University School of Medicine, Tokyo, Japan; 2 Department of Regenerative Medicine and Advanced Cardiac Therapeutics, Keio University School of Medicine, Tokyo, Japan; 3 Calcium Oscillation Project, ICORP-SORST, Japan Science and Technology Agency, Saitama, Japan; 4 Laboratory for Developmental Neurobiology, Brain Science Institute (BSI), RIKEN, Saitama, Japan; Harvard Medical School, United States of America

## Abstract

**Background:**

Inositol 1,4,5-trisphosphate receptors (IP_3_R1, 2, and 3) are intracellular Ca^2+^ release channels that regulate various vital processes. Although the ryanodine receptor type 2, another type of intracellular Ca^2+^ release channel, has been shown to play a role in embryonic cardiomyocytes, the functions of the IP_3_Rs in cardiogenesis remain unclear.

**Methodology/Principal Findings:**

We found that *IP_3_R1^−/−^-IP_3_R2^−/−^* double-mutant mice died *in utero* with developmental defects of the ventricular myocardium and atrioventricular (AV) canal of the heart by embryonic day (E) 11.5, even though no cardiac defect was detectable in *IP_3_R1^−/−^* or *IP_3_R2^−/−^* single-mutant mice at this developmental stage. The double-mutant phenotype resembled that of mice deficient for calcineurin/NFATc signaling, and NFATc was inactive in embryonic hearts from the double knockout-mutant mice. The double mutation of *IP_3_R1/R2* and pharmacologic inhibition of IP_3_Rs mimicked the phenotype of the AV valve defect that result from the inhibition of calcineurin, and it could be rescued by constitutively active calcineurin.

**Conclusions/Significance:**

Our results suggest an essential role for IP_3_Rs in cardiogenesis in part through the regulation of calcineurin-NFAT signaling.

## Introduction

Intracellular Ca^2+^ signaling is crucial for cardiac functions [1]. Two types of Ca^2+^ release channels on the sarcoplasmic/endoplasmic reticulum (SR/ER) serve to regulate Ca^2+^ release from intracellular Ca^2+^ stores: the ryanodine receptor (RyR) and inositol 1,4,5-trisphosphate receptor (IP_3_R). RyR is mainly required for physiologic excitation–contraction coupling in the heart, whereas IP_3_R mediates Ca^2+^ mobilization, in response to IP_3_ produced by phospholipase C activation, not only in most non-excitable cells but also in excitable cells including cardiomyocytes [2]. There have been identified three subtype of IP_3_Rs (IP_3_R1, IP_3_R2 and IP_3_R3), derived from three distinct genes in mammals [3], [4]. We previously generated mice that lacked IP_3_R1, IP_3_R2 and IP_3_R3 by disrupting the corresponding gene within the first exon [5], [6], and reported the cerebellar phenotype of *IP_3_R1^−/−^* mice [5] and the pancreatic phenotype of *IP_3_R2^−/−^-IP_3_R3^−/−^* mice [6], thereby demonstrating the specific and redundant roles of IP_3_Rs in organ development and function. Regarding the heart, each *IP_3_R1, IP_3_R2,* and *IP_3_R3* single-mutant mouse showed normal cardiogenesis, in contrast to the ryanodine receptor type 2 single-mutant mouse, which showed embryonic lethality owing to dysfunction of the SR in the embryonic cardiomyocyte [7].

Extracellular ligands binding to many receptors, including G-protein coupled receptors and tyrosine-kinase coupled receptors, lead to a transient release of Ca^2+^ from ER/SR, through IP_3_Rs. IP_3_-induced Ca^2+^ release concurrently results in depletion of intracellular Ca^2+^ store, which triggers Ca^2+^ release activated Ca^2+^ (CRAC) channels [8]. Subsequent increase of cytosolic [Ca^2+^] through CRAC channels activates several Ca^2+^- binding proteins, including calcineurin, which in turn dephosphorylates and induces the nuclear localization of the nuclear factor of activated T cells (NFAT) transcription complexes [9]. During heart development, NFATc1 is expressed in the endocardium of the AV canal that will make up the endocardial cushion [10]. NFATc1 knockout embryos show abnormal valvulogenesis [11], [12], while NFATc2/3/4 triple knockout embryos and calcineurin-deficient embryos demonstrate impaired endocardial cushion formation, thinning of ventricular myocardium and dysregulation of vascular development [10], [13], [14]. To determine the function of the intracellular Ca^2+^ signaling cascade via IP_3_Rs in the embryonic hearts, here we generated and analyzed IP_3_R1 and IP_3_R2-deficient mice. Our findings support an essential redundant role of IP_3_R1 and IP_3_R2 during cardiogenesis, possibly implicating the calcineurin/NFAT signaling pathway.

## Results

### Overlapping Expression Patterns of IP_3_R1 and IP_3_R2 in Embryonic Hearts

**Figure 1:**
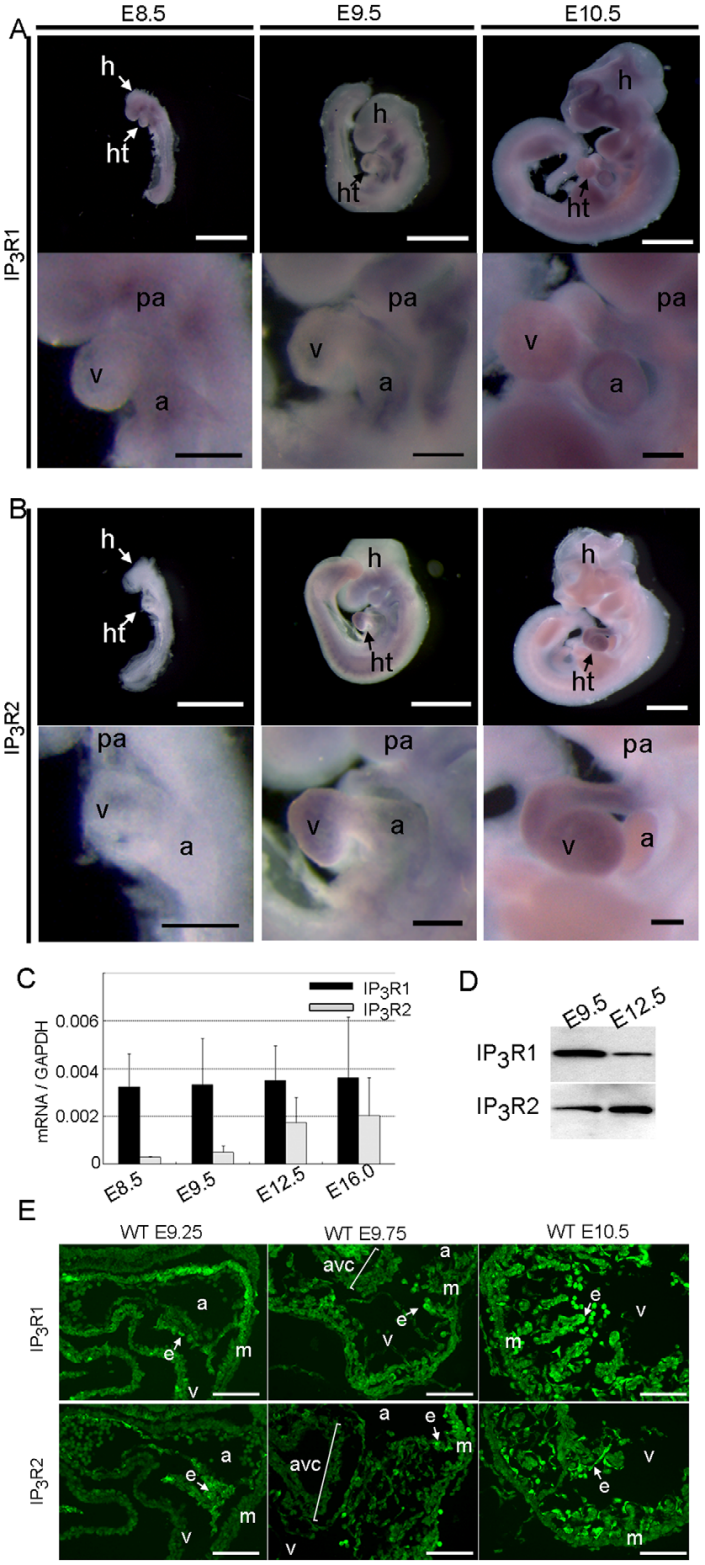
Both IP_3_R1 and IP_3_R2 are expressed in the embryonic heart. (*A, B*) Whole-mount *in situ* hybridization using IP_3_R1 (*A*) and IP_3_R2 (*B*) antisense riboprobes at E8.5, E9.5, and E10.5. Whole-mount views (upper panels) and close-up views of the hearts (lower panels) are shown. Scale bars: 1 mm (whole-mount views) and 0.2 mm (heart close-up views). (*C*) Quantitative RT-PCR of IP_3_R1 (black bars) and IP_3_R2 (gray bars) using total RNA samples extracted from embryonic hearts at E8.5 to E16.0. Error bars indicate standard deviations. (*D*) Western blot of embryonic hearts with anti-IP_3_R1 (18A10) and anti-IP_3_R2 (KM1083) antibodies. Lysates (10 µg) of hearts at E9.5 and E12.5 were analyzed. The protein contents of the heart extracts were determined by the Bradford method. (*E*) The transverse sections of the wildtype embryos at E9.25, E9.75 and E10.5 were immunostained with the anti-IP_3_R1 (upper panels) and anti-IP_3_R2 (lower panels) antibodies. Scale bars: 0.1 mm. a, atrium; avc, atrioventricular canal; e, endocardium; h, head; ht, heart; m, myocardium; pa, pharyngeal arch; v, ventricle.

Firstly, we examined the normal pattern of expression of the IP_3_Rs by RNA *in situ* hybridization. Consistent with a previous report [15], expression of IP_3_R1 mRNA was detected at embryonic day (E)8.5 in the heart, where it was enhanced in the posterior part of the primitive heart, including the atrium (Fig. 1*A*). IP_3_R1 mRNA expression extended to the ventricles through the AV canal at E9.5–10.5 (Fig. 1*A*). In contrast, IP_3_R2 mRNA expression was not detected at E8.5 but was detected at E9.5 throughout the heart, including the atrium, AV canal, and ventricles (Fig. 1*B*). These expression signals were not detected by sense riboprobes as a control (data not shown). Quantitative RT-PCR using total RNA extracted from hearts at E8.5 to E16.0 and western blot analysis of proteins at E9.5 and E12.5 indicated that the IP_3_R1 and IP_3_R2 transcripts and proteins were expressed at significant levels in the developing hearts (Fig. 1*C* and *D*), consistent with the results of the RNA *in situ* hybridization experiments. We performed an immunohistochemical analysis on sections of the heart at E9.25, E9.75 and E10.5 to determine the cell types in the embryonic heart that express the IP_3_R proteins. At E9.25, IP_3_R1 was expressed in both endocardial cells and myocardial cells, whereas IP_3_R2 was expressed dominantly in endocardial cells (Fig. 1*E*). Co-expression of IP_3_R1 and IP_3_R2 was observed in the endocardial cells of the AV canal (Fig. 1*E*). At E9.75 to E10.5, the expression of IP_3_R2 expanded to the myocardium and IP_3_R1 and IP_3_R2 were co-expressed not only in the endocardial cells but also in the myocardial cells (Fig. 1*E*). These expression signals with anti- IP_3_R1 and anti- IP_3_R2 antibodies were not detected in the sections of the *IP_3_R1^−/−^-IP_3_R2^−/−^* double-mutant embryos as a control (data not shown).

### Cardiac Defects in *IP_3_R1^−/−^-IP_3_R2^−/−^* Double-Mutant Mice

**Figure 2:**
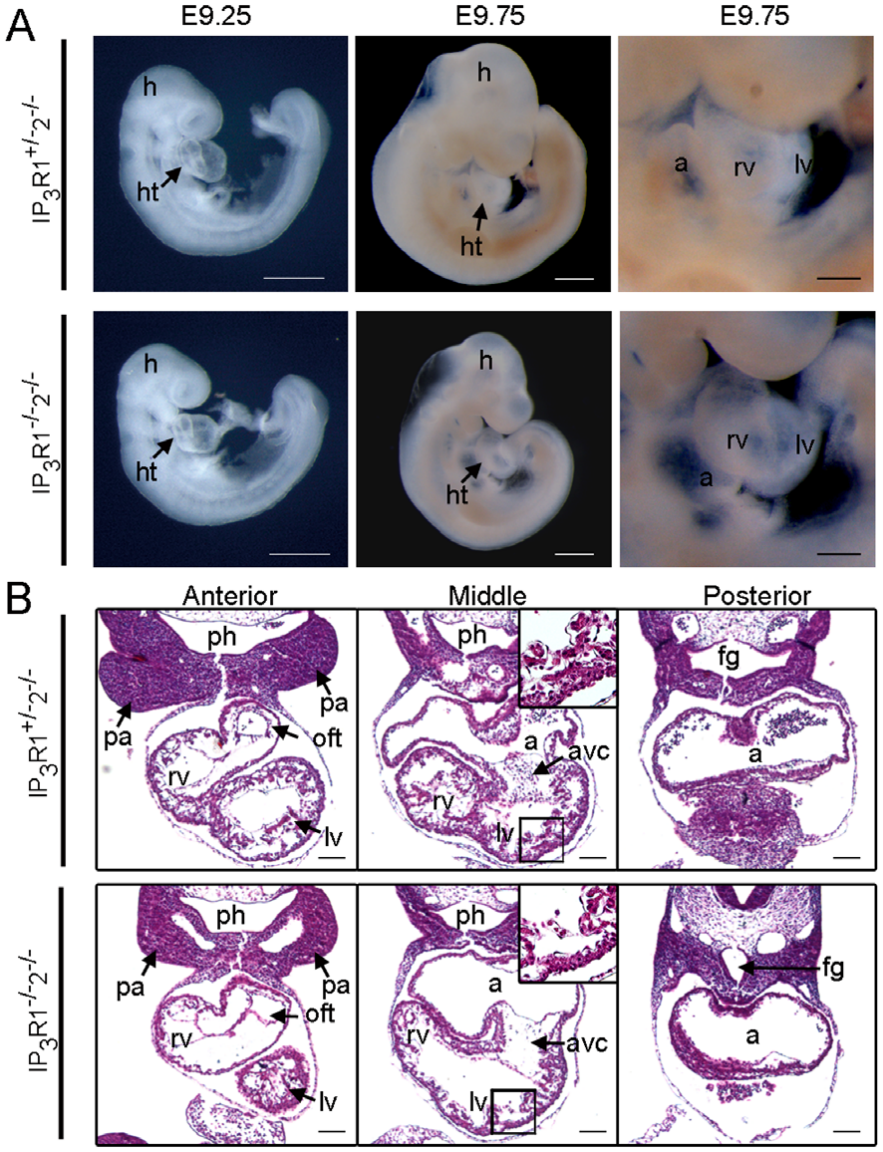
Cardiac defects in *IP_3_R1^−/−^-IP_3_R2^−/−^* mice. (*A*) The morphologies of the embryos and developing hearts of *IP_3_R1^+/−^-IP_3_R2^−/−^* (upper panels) and *IP_3_R1^−/−^-IP_3_R2^−/−^* (lower panels) mice at E9.25 and at E9.75. Scale bars, 0.5 mm (whole-mount views) and 0.2 mm (heart close-up views). (*B*) Hematoxylin and eosin-stained transverse sections of the anterior, middle and posterior segments of the hearts of *IP_3_R1^+/−^-IP_3_R2^−/−^* (upper panels) and *IP_3_R1^−/−^-IP_3_R2^−/−^* (lower panels) mice at E9.75. The insets in the middle panels are higher-magnification images of the boxed areas. Scale bars, 0.2 mm. a, atrium; avc, atrioventricular canal; fg, foregut; h, head; ht, heart; lv, left ventricle; oft, outflow tract; pa, pharyngeal arch; ph, pharynx; rv, right ventricle.

To explore further the roles of the IP_3_Rs in cardiac development, we delineated the cardiac phenotype of the IP_3_R mutant mouse. *IP_3_R1^−/−^-IP_3_R2^−/−^* double-mutant mice showed embryonic lethality by E11.5 (see supporting information (SI) Table S1) with heart defects, while either *IP_3_R1^+/−^-IP_3_R2^−/−^* or *IP_3_R1^−/−^-IP_3_R2^+/+^* mouse developed normally through E11.5. The *IP_3_R1^−/−^-IP_3_R2^−/−^* double-mutant mice began to show growth retardation at E9.5, although they appeared normal by E9.25 and had regular cardiac contractions comparable to those of wild-type. Detailed analysis of mouse hearts at E9.75 showed that the ventricles of *IP_3_R1^−/−^-IP_3_R2^−/−^* mice were more transparent than those of *IP_3_R1^+/−^-IP_3_R2^−/−^* mice, even though comparable 29 pairs of somites were developed, cardiac looping occurred normally, and morphologic atria and ventricles were apparent in both *IP_3_R1^−/−^-IP_3_R2^−/−^* mice and *IP_3_R1^+/−^-IP_3_R2^−/−^* mice (Fig. 2*A*). Histological analysis revealed thin myocardial walls and poor trabeculation in the ventricles of *IP_3_R1^−/−^-IP_3_R2^−/−^* mice at E9.75 (Fig. 2*B*). Together with the ventricular abnormalities, hypocellularity in the cushion of AV canal was documented in the *IP_3_R1^−/−^-IP_3_R2^−/−^* hearts (Fig. 2*B*). Taken together with the evidence of overlapping expression of *IP_3_R1* and *IP_3_R2* in the developing ventricles and AV canal at E9.5 or later, these results suggest that these two genes play redundant roles in cardiogenesis. Although histologic abnormalities were noted, analysis using electron microscopy found no structural defects in the subcellular organelles, in the *IP_3_R1^−/−^-IP_3_R2^−/−^* hearts at E9.5 (Fig. S1).

We performed a molecular analysis to investigate the association between the *IP_3_R1^−/−^-IP_3_R2^−/−^* double-mutant phenotype and the differentiation of atrial and ventricular cardiomyocytes. Nkx2.5, Mlc2a, and Mlc2v are the earliest markers of cardiomyocyte differentiation in the atrium and ventricle during cardiogenesis [16], [17], [18]. These markers were expressed at normal levels in *IP_3_R1^−/−^-IP_3_R2^−/−^* hearts at E9.5 (Fig. S2). Markers for the right ventricle (Hand2 [19]), left ventricle (Hand1 [19]) and both ventricles (Hrt2 [20]) were also expressed normally in *IP_3_R1^−/−^-IP_3_R2^−/−^* hearts at E9.5 (Fig. S2). These results suggest that cardiomyocyte differentiation and the specification of both ventricles occur normally in *IP_3_R1^−/−^-IP_3_R2^−/−^* hearts despite poor formation of the ventricles.

In spite of the well-developed organs other than hearts in *IP_3_R1^−/−^-IP_3_R2^−/−^* embryos the early embryonic lethality of *IP_3_R1^−/−^-IP_3_R2^−/−^* mice and a previous report on the requirement for certain isotypes of phospholipase C_δ_ that produce IP_3_ for placentation [21], [22] led us to investigate the possibility of placental defects in *IP_3_R1^−/−^-IP_3_R2^−/−^* mice. Histologic analysis revealed that the embryonic vessels elongated and contact with the maternal blood vessels in the *IP_3_R1^−/−^-IP_3_R2^+/−^* or *IP_3_R1^+/−^-IP_3_R2^−/−^* placentas at E9.5. The area of the labyrinth layer, which comprises a network of embryonic and maternal blood vessels, was significantly smaller in the *IP_3_R1^−/−^-IP_3_R2^−/−^* placenta than that in the *IP_3_R1^+/−^-IP_3_R2^−/−^* placenta at E9.5 (Fig. S3).

### Downregulated Cell Proliferation Activity in *IP_3_R1^−/−^-IP_3_R2^−/−^* Double-Mutant Ventricles

**Figure 3:**
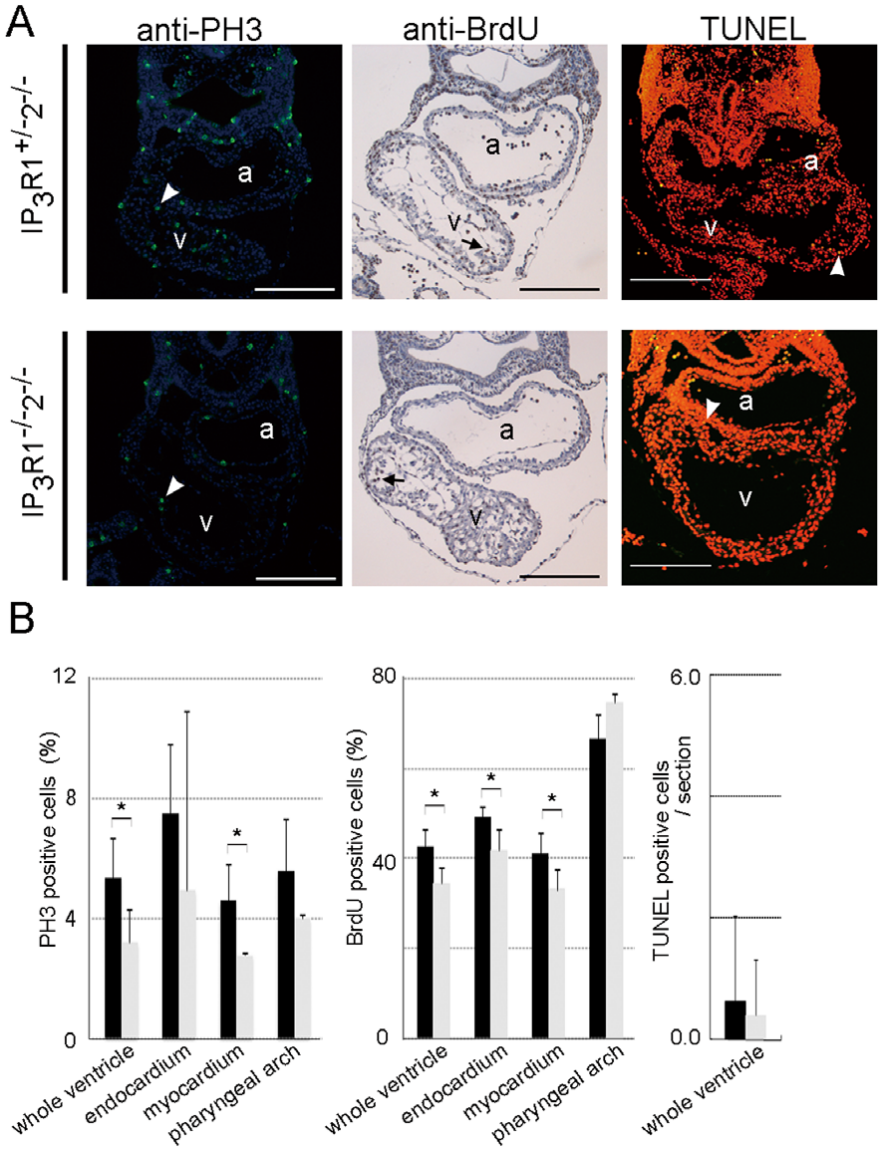
IP_3_R1 and IP_3_R2 redundantly regulate cardiomyocyte proliferation in the developing ventricles. (*A*) Transverse sections of E9.5 *IP_3_R1^+/−^-IP_3_R2^−/−^* (upper panels) and *IP_3_R1^−/−^-IP_3_R2^−/−^* (lower panels) embryos that were immunostained with the anti-phospho-histone H3 (PH3) antibody, immunostained with the anti-BrdU antibody after injection of BrdU, and subjected to the TUNEL assay. The green signals (white arrowheads) in the left panels, brown signals in the middle panels (arrows) and yellow signals (white arrowheads) in the right panels indicate the nuclei of the PH3-, BrdU-, and TUNEL-positive cells, respectively. Scale bars, 0.2 mm. a, atrium; v, ventricle. (*B*) The left and middle graphs show the percentages of proliferating cells in the whole ventricles, the endocardium and myocardium of the ventricles, and the pharyngeal arches of *IP_3_R1^−/−^-IP_3_R2^−/−^* mutants (gray bars), compared with those of *IP_3_R1^+/−^-IP_3_R2^−/−^* controls (black bars) (*P<0.05, n = 3). The right graph shows the number of apoptotic cells (*P* = 0.45, n = 7). Error bars indicate standard deviations.

Next, we looked in the ventricles of *IP_3_R1^−/−^-IP_3_R2^−/−^* mice at E9.5 for cell proliferation markers using the anti-phospho-histone H3 (PH3) antibody and 5-bromo-2′-deoxy-uridine (BrdU), and performed the TUNEL assay for apoptosis (Fig. 3 *A* and *B*). The numbers of PH3-positive cells and BrdU-incorporated cells were lower in the *IP_3_R1^−/−^-IP_3_R2^−/−^* ventricular endocardium and myocardium than in those of *IP_3_R1^+/−^-IP_3_R2^−/−^* hearts, whereas the numbers of proliferating cells in the pharyngeal arch and the numbers of apoptotic signals detected by the TUNEL assay were similar between *IP_3_R1^−/−^-IP_3_R2^−/−^* and *IP_3_R1^+/−^-IP_3_R2^−/−^* embryos (Fig. 3*A* and *B*). These results indicate that the redundant functions of IP_3_R1 and IP_3_R2 are essential for ventricular cell proliferation. The *IP_3_R1^−/−^-IP_3_R2^−/−^* mouse phenotype is similar to that of mice lacking both Ca^2+^-sensitive transcription factors, NFATc3 and NFATc4, where the proliferation of the ventricular cells is considerably reduced [14].

### IP_3_R1 and IP_3_R2 Redundantly Control Endocardial Cushion Development through Calcineurin/NFATc Signaling

**Figure 4:**
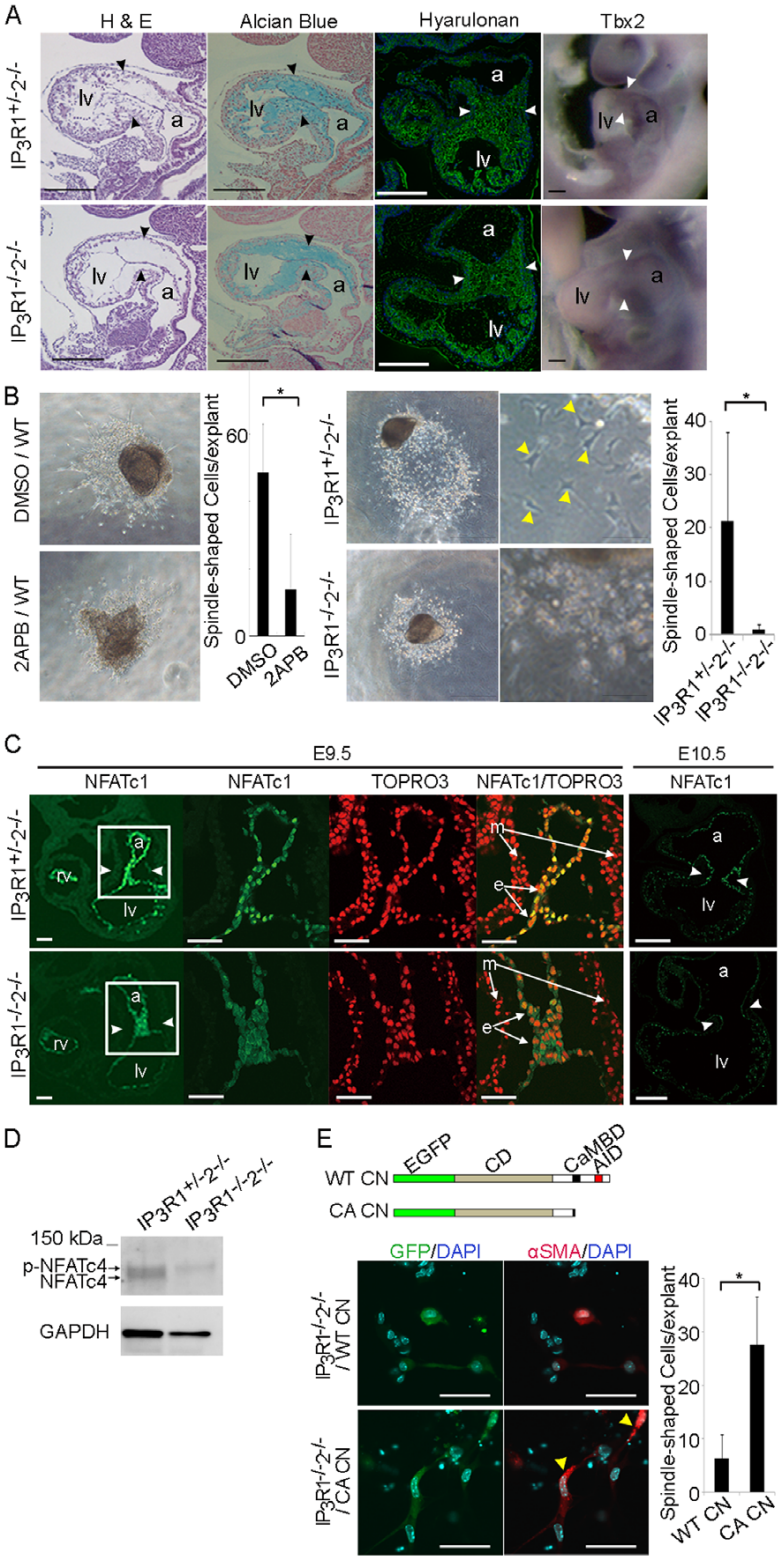
IP_3_Rs are essential for EMT through calcineurin activity during endocardial cushion development in mice. (*A*) Hematoxylin and eosin staining, alcian blue staining, staining with biotinylated hyaluronan binding protein and whole-mount *in situ* hybridization for a marker of the atrioventricular (AV) myocardium (Tbx2) of *IP_3_R1^+/−^-IP_3_R2^−/−^* (upper panels) and *IP_3_R1^−/−^-IP_3_R2^−/−^* (lower panels) embryos at E9.5. Mesenchymal cells are absent from the *IP_3_R1^−/−^-IP_3_R2^−/−^* AV cushion (arrowheads), whereas the staining levels for alcian blue (blue signals), hyaluronan (green signals) and Tbx2 (purple signals) are not altered. Scale bars, 0.1 mm. (*B*) *In vitro* EMT assay using the endocardial cushion from the AV canal. The left panels show that treatment of wild-type (WT) cushion explants with the IP_3_R inhibitor 2APB inhibits the outgrowth of spindle-shaped cells as compared with the control treatment (DMSO). The right panels show the *IP_3_R1^+/−^-IP_3_R2^−/−^* and the *IP_3_R1^−/−^-IP_3_R2^−/−^* AV cushion explants. The higher magnification views of the explants are shown besides. The spindle-shaped cells migrating into collagen gel are indicated with yellow arrowheads. The number of the spindle-shaped migrating cells is significantly lower in the culture that contains 2APB (left graph) and in the culture from the *IP_3_R1^−/−^-IP_3_R2^−/−^* AV cushion (right graph) (*P<0.05, n = 3). Error bars indicate standard deviations. (*C*) Transverse sections at the level of the AV canal stained with the anti-NFATc1 antibody and TOPRO3 show impairment of translocation of NFATc1 into nuclei at E9.5. The expression level of NFATc1 is decreased at E10.5 in *IP_3_R1^−/−^-IP_3_R2^−/−^* hearts. Scale bars, 0.05 mm. Arrowheads indicate the AV canal and higher-magnification images of the boxed area. (*D*) Western blot analysis with anti-NFATc4 antibody using heart lysates of the wildtype and the *IP_3_R1^−/−^-IP_3_R2^−/−^* embryos at E9.75. Blotting with anti-GAPDH antibody on each lane was used as a loading control. The inactive form of NFATc4 (p-NFATc4) remained and active form (NFATc4) was reduced in *IP_3_R1^−/−^-IP_3_R2^−/−^* hearts compared to *IP_3_R1^+/−^-IP_3_R2^−/−^* hearts. (*E*) The structures of the wildtype calcineurin (WT CN) and the constitutively active form of calcineurin (CA CN) cDNA are shown. Infection with CA CN cDNA resulted in significant increase in the number of the spindle-shaped and α smooth muscle actin (αSMA)-positive cells (yellow arrowheads) in the culture of *IP_3_R1^−/−^-IP_3_R2^−/−^* AV cushion explants (*P<0.05, n = 4). a, atrium; e, endocardium; lv, left ventricle; m, myocardium, rv, right ventricle; v, ventricle.

Histological analyses of the developing hearts were performed using alcian blue staining, which detects acidic mucosubstances, as well as staining with biotinylated hyaluronan binding protein for visualizing hyaluronan production (Fig. 4*A*). These analyses revealed the absence of mesenchymal cells in the cushion of the AV canal of *IP_3_R1^−/−^-IP_3_R2^−/−^* mice at E9.5, whereas the volume and content of the cardiac jelly in the *IP_3_R1^−/−^-IP_3_R2^−/−^* hearts were comparable with those in the *IP_3_R1^+/−^-IP_3_R2^−/−^* hearts. Expression of the transcription factor Tbx2 was also normal in the AV canals of *IP_3_R1^−/−^-IP_3_R2^−/−^* mice, which suggests that specification and differentiation of the AV canal is unaffected in the mutant mice (Fig. 4*A*). These findings led us to hypothesize that IP_3_R1 and IP_3_R2 were required for the epithelial-mesenchymal transformation (EMT) in the developing AV cushion. To determine whether IP_3_R-mediated Ca^2+^ signaling could affect the process of EMT in the AV cushion, we performed an *in vitro* EMT assay [23]. The E9.5 wild-type AV canal tissues were explanted and treated with 2-aminoethoxydiphenyl borate (2APB) onto a collagen gel, which is a non-selective membrane-permeable inhibitor of IP_3_Rs and CRAC channels [24]. After 24 h of treatment with 2APB, EMT was inhibited, resulting in a significant decrease in the number of cells that transformed and migrated into the collagen gel compared with control explants treated with DMSO (Fig. 4*B*). Similarly, EMT was significantly inhibited in the AV canal explants from *IP_3_R1^−/−^-IP_3_R2^−/−^* embryo at E9.5 (Fig. 4*B*).

Of the several signaling pathways implicated in AV cushion and myocardial development, we focused on calcineurin/NFAT signaling because of the similarities noted between the phenotypes of the *IP_3_R1^−/−^-IP_3_R2^−/−^* mice and calcineurin- or *NFATc*-deficient mice [10], [13], [14]. To determine whether IP_3_R1 and IP_3_R2 regulate intracellular Ca^2+^ signaling upstream of calcineurin/NFATc, we examined the cellular localization of NFATc1 in the AV canals of *IP_3_R1^−/−^-IP_3_R2^−/−^* mice. Immunohistochemical analysis revealed that NFATc1 failed to translocate into the nuclei of the endocardial cells in the AV canals of *IP_3_R1^−/−^-IP_3_R2^−/−^* mice at E9.5, and that the expression of NFATc1 was downregulated by E10.5 (Fig. 4*C*). Nuclear translocation of NFATc1 appeared normal at E9.25 but was inhibited from E9.5 (Fig. S4). Next, we examined the activation of NFATc4 by western blot analysis using the lysates from *IP_3_R1^−/−^-IP_3_R2^−/−^* hearts at 9.75. The quantity of the dephosphorylated form of NFATc4 was decreased in the *IP_3_R1^−/−^-IP_3_R2^−/−^* heart compared to the *IP_3_R1^+/−^-IP_3_R2^−/−^* heart (Fig. 4*D*). These observations are reminiscent of those made for calcineurin B*/* mice, in which the activation of calcineurin B is disrupted [13]. To provide further evidence for the possible interaction between IP_3_Rs and calcineurin/NFATc signaling, we tested whether constitutively active calcineurin could rescue the phenotype of *IP_3_R1^−/−^-IP_3_R2^−/−^*. Infection of cytomegalovirus-associated constitutively active calcineurin, which can be activated independently of Ca^2+^ increase by deletion of its calmodulin binding domain and autoinhibitory domain [25], significantly restored the EMT activity in *IP_3_R1^−/−^-IP_3_R2^−/−^* AV explants (Fig. 4*E*). Taken together, our results indicate that IP_3_R1 and IP_3_R2, at least in part, redundantly activate and maintain calcineurin/NFATc signaling.

**Figure 5:**
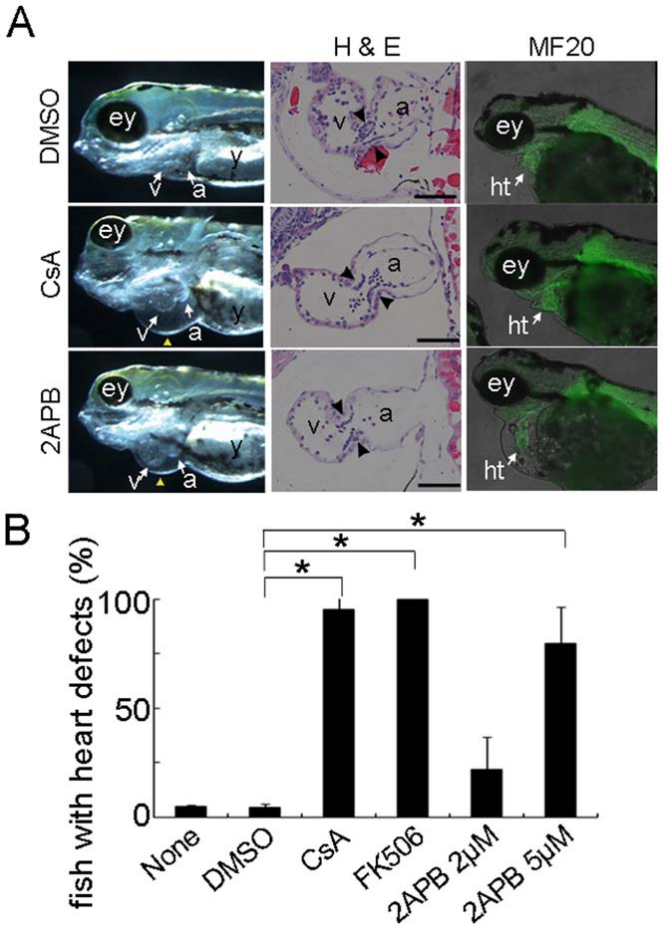
Inhibition of IP_3_Rs and calcineurin results in a common developmental defect in zebrafish hearts. (*A*) The left panels show the gross abnormalities in the hearts of zebrafish treated with DMSO, CsA (calcineurin inhibitor), and 2APB (IP_3_R inhibitor). Atrioventricular regurgitation is induced by the addition of CsA and 2APB, resulting in heart failure with pericardial swelling (yellow arrowheads). The middle panels show the histologic abnormalities including a decrease in the number of cushion cells in the atrioventricular valves (arrowheads). The right panels show the expression of sarcomeric protein MF20 in zebrafish hearts that did not alter with CsA- or 2APB-treatment. (*B*) The significant increase in the number of zebrafish with pericardial swelling following the addition of CsA, FK506, or 2APB, is indicated in the graph (*P<0.05, n = 3). Error bars indicate standard deviations. a, atrium; ey, eye; ht, heart; v, ventricle; y, yolk.

To investigate further whether IP_3_R-mediated Ca^2+^ signaling is required for AV cushion development, we utilized a relatively simple model of heart development in zebrafish treated with an inhibitor of IP_3_R (Fig. 5*A*). As described previously [10], treatment with the calcineurin inhibitors cyclosporine A (CsA) or FK506 between 24 h and 33 h postfertilization (hpf) affects AV cushion development and heart valve formation in zebrafish. Intriguingly, treatment with 2APB between 24 hpf and 33 hpf disrupted AV cushion development in zebrafish (Fig. 5*A*) in a dose-dependent fashion (Fig. 5*B*). This results in a massive regurgitation of the AV valve and heart failure with pericardial effusion reminiscent of treatment with CsA (Fig. 5*A* and Video S1). Although myocardial development appeared normal by staining of the sarcomeric protein MF20, disruption of AV canal cushion development, and possibly decreased myocardial function, was common in CsA-treated and 2APB-treated zebrafish (Fig. 5*A* and *B*). These data further support that IP_3_R-mediated signaling is essential for AV cushion development, possibly implicating the calcineurin/NFAT signaling across species.

## Discussion

In the present study, we used a series of molecular, pharmacologic and genetic manipulations in mice and zebrafish to demonstrate that IP_3_Rs are involved in the local control of Ca^2+^ that is necessary to activate the calcineurin-NFATc signaling pathway required for cardiogenesis. To date, it has been shown that calcineurin/NFATc signaling has an important role for AV cushion formation, valvulogenesis, and cardiomyocyte development [10], [11], [12], [14], however, the process by which this signaling is activated by Ca^2+^ has not been elucidated. In *Xenopus* embryos, NFAT is a downstream effecter of the IP_3_-Ca^2+^ signal during dorsoventral axis formation [26]. In mammals, IP_3_Rs act upstream of calcineurin/NFAT signaling in T lymphocytes *in vitro* [27]. In this study, we have indentified that IP_3_R1 and IP_3_R2 may exist, at least in part, upstream of calcineurin/NFATc signaling during heart development. Our data provide new insights into the way how Ca^2+^ signals regulate Ca^2+^-binding molecules essential for cardiogenesis, such as the Ca^2+^ dependent phosphatase, calcineurin.

Here, we have shown several lines of findings that support that the IP_3_R-mediated Ca^2+^ signaling may be implicated in the calcineurin/NFATc signaling pathway although it is difficult to clarify whether IP_3_Rs may directly activate calcineurin during cardiogenesis. We showed that NFATc1 and NFATc4 were not activated in *IP_3_R1^−/−^-IP_3_R2^−/−^* mice at E9.5 (Fig. 4*C* and *D*), and that the expression of NFATc1 was downregulated by E10.5 (Fig. 4*C*), using immunohistological and biochemical experiments. Moreover, we showed that the disturbance of endocardial cushion development in *IP_3_R1^−/−^-IP_3_R2^−/−^* embryos could be rescued by addition of constitutively active calcineurin (Fig. 4*E*). These data indicate that IP_3_R1 and IP_3_R2 may, directly or indirectly, function redundantly in the activation of calcineurin/NFAT signaling. Vascular endothelial growth factor (VEGF), inducing NFATc1 nuclear translocation, markedly increases the proliferation of valve endothelial cells *in vitro* [28]. The VEGF receptor tyrosine kinase activating phospholipase Cγ- IP_3_ signal triggers intracellular Ca^2+^ increase [29], suggesting that VEGF might be a candidate of extracellular signals upstream of IP_3_Rs-NFATc1 activation in the endocardium. Failure of nuclear translocation and downregulation of NFATc1 is probably due to disruption of some of the following positive feedback mechanisms: 1) NFATc1-binding to the promoter/enhancer of the *NFATc* gene [30]; 2) activation of calcineurin transcription by NFATc [31]; and 3) induction of the *IP_3_R1* gene as previously documented in neurons [32].

We also showed that the phenotype of *IP_3_R1^−/−^-IP_3_R2^−/−^* embryos involved that of the *NFATc2/3/4* knockout mice [10], [13], [14] and was much more severe than that of the *NFATc1* null mice [11], [12]. Therefore, it is likely that IP_3_R-mediated Ca^2+^ signaling during cardiogenesis regulates NFATc family members in addition to NFATc1 as well as unknown downstream targets other than the calcineurin/NFATc signaling pathway. It is also unclear whether endocardial or myocardial cells are primarily affected in the *IP_3_R1^−/−^-IP_3_R2^−/−^* embryos. In characterizing the role of this pathway in heart development, our study revealed the co-expression and involvement of IP_3_R1 and IP_3_R2 in the development of both endocardial and myocardial cells. Unequivocal answer about the functions of IP_3_R1 and IP_3_R2 for cardiac development awaits further study of cell-specific conditional knockout mice.

Although our experiments in AV explants and in zebrafish using 2APB support our hypothesis led by our experiments using *IP_3_R1^−/−^-IP_3_R2^−/−^* mice, there is a limitation for using 2APB. Because 2APB is non-selective inhibitor, it is a potent CRAC channel inhibitor in addition to having an inhibitory effects on not only IP_3_R1 and IP_3_R2, but also other subtype of IP_3_R [33]. Unfortunately, there is no selective IP_3_R inhibitor presently available. The observed defect in AV explants and in zebrafish with 2APB should include the possible effects of 2APB on CRAC channel activity, although AV explants from *IP_3_R1^−/−^-IP_3_R2^−/−^* mice showed similar defect. There also is a limitation to determine the cause of death in the *IP_3_R1^−/−^-IP_3_R2^−/−^* mice in this study. The placental defect, in conjunction with the cardiac defect, are likely to account for embryonic lethality in these mice, however, we could neither correctly estimate impact of each defect nor rule out defects of other organs. Further strategy for inactivation of the specific IP_3_R in specific tissues and organs would be required to overcome these limitations appeared in this study.

The DSCR1/MCIP1 gene encoding a calcineurin inhibitor is located on human chromosome 21 and a reduction of NFATc activity is associated with a 1.5-fold increase in gene dosage of DSCR1 and many of the features of Down (Trisomy 21) syndrome, including AV canal defects [34]. The balance between Ca^2+^-sensitive positive (e.g., IP_3_Rs) and negative (e.g., DSCR1) regulation of NFATc may be crucial for organogenesis. Understanding the novel roles of IP_3_R-mediated Ca^2+^ signaling opens new avenues of research regarding the molecular embryology of the heart, which may lead to regenerative interventions for patients with congenital heart defects.

## Materials and Methods

### Animals

*IP_3_R1/2* double-knockout mice were generated by intercrossing *IP_3_R1^+/−^-IP_3_R 2^−/−^* paired mice [5], [6]. Genomic DNA samples prepared from tail biopsies or yolk sacs were subjected to PCR for genotyping, as described previously [5], [6]. Zebrafish (*Danio rerio*) embryos were obtained from the natural spawning of a wild-type ABK line. CsA (50 µg/mL) or 2APB (5 µM) was added between 24 hpf and 33 hpf, and the zebrafish were harvested at 72 hpf for analysis [10]. All experimental procedures and protocols were approved by the animal care and use committees of Keio University (the approval number 09122-(3)) and conformed to the National Institutes of Health *Guidelines for the Care and Use of Laboratory Animals*.

### Western Blotting, Quantitative RT-PCR, Whole-Mount *in situ* Hybridization, and Immunohistochemistry

Total heart extracts (10 µg) from E9.5 and E12.5 mice were subjected to 5% SDS-PAGE and analyzed by western blotting with anti-IP_3_R1 (18A10) [35] and anti-IP_3_R2 (KM1083) antibodies [36]. For quantitative RT-PCR analysis of IP_3_R1 and IP_3_R2 expression, cDNAs were synthesized using the High Capacity cDNA Archive kit (Applied Biosystems) from total RNA samples extracted from embryonic hearts or placentas using the RNeasy kit (Qiagen). These cDNAs were used as templates in a TaqMan Real-Time PCR with the ABI 7500 Real-Time PCR system (Applied Biosystems). The data are normalized to the levels obtained for *GAPDH*. The TaqMan probes used for *IP_3_R1* and *IP_3_R2* were Mm00439917_m1 and Mm00444937_m1 (Applied Biosystems), respectively. The whole-mount RNA *in situ* hybridization and immunohistochemistry were performed as previously described [13], [37]. The antibodies used for immunohistochemistry on the sections were: mouse anti-NFATc1 (7A6) monoclonal antibody (1∶100; Santa Cruz Biotechnology), anti-phospho-histone H3 (Ser10) antibody (1∶100; Upstate), anti-BrdU antibody (1∶75; Becton Dickinson), anti-NFATc4 (H-74) antibody (1∶250; Santa Cruz Biotechnology) and anti-α smooth muscle actin (1A4) antibody (1∶400; Sigma).

### EMT Assay

The EMT assay was performed as reported previously [23]. Endocardial cushions from the atrioventricular canal were explanted onto rat-tail collagen gel (BD Biosciences). After 24 h of incubation with 2APB, which is a non-selective inhibitor of IP_3_Rs [24], or with DMSO as a control, the total number of mesenchymal cells in each dish was counted. AV canal explants were infected using inactivated adenovirus harboring the gene encoding cytomegalovirus-driven constitutively active calcineurin [25] (Adeno-X ViraTrak Expression System 2, Clontech). After overnight incubation to allow attachment, each explant was incubated with 6.4×10^4^ viral particles in culture medium. Whereupon, the AV explants were cultured for an additional 5 days and then assessed as indicated.

### Statistical Analysis

All data are expressed as mean ± s.d. (n≥3). Statistical analysis was performed using the Student's *t*-test. The results shown are representative of more than three independent experiments.

## Supporting Information

Materials and Methods S1 (0.03 MB DOC)

Table S1 Genotype Distributions of Embryos from *IP_3_R1^+/−^-IP_3_R2^−/−^* Intercrosses.
(0.03 MB DOC)

Figure S1 The ultrastructures of the subcellular organelles of the *IP_3_R1^−/−^-IP_3_R2^−/−^* mice are comparable with those of the *IP_3_R1^+/−^-IP_3_R2^−/−^* mice. Scale bars, 1 µm. g, golgi; m, mitochondrion; mf, myofilament; n, nucleus; rer, rough endoplasmic reticulum; sr, sarcoplasmic reticulum.
(1.74 MB TIF)

Figure S2 Expression of site-specific markers in embryonic hearts. Whole-mount *in situ* hybridization images of the left- or right-side of the hearts of E9.5 *IP_3_R1^+/−^-IP_3_R2^−/-^* (upper panels) and *IP_3_R1^−/−^-IP_3_R2^−/−^* (lower panels) embryos. The expression patterns of Nkx2.5, MLC2a, and MLC2v (earliest markers of the embryonic heart), and of Hand2, Hand1 and Hrt2 (markers of the right, left, and both ventricles, respectively) are shown. Scale bars, 0.2 mm. a, atrium; lv, left ventricle; oft, outflow tract; pa, pharyngeal arch; rv, right ventricle.
(5.26 MB TIF)

Figure S3 Cross-sections of E9.5 placentas from *IP_3_R1^−/−^-IP_3_R2^+/−^* (upper panels), *IP_3_R1^+/−^-IP_3_R2^−/−^* (middle panels) and *IP_3_R1^−/−^-IP_3_R2^−/−^* (lower panels) mutant mice. The widths of the labyrinth area indicated in parentheses. Higher-magnification images of the boxed areas are shown in the right panels. Scale bars, 0.5 mm. The graph shows quantification of the labyrinth areas of the *IP_3_R1^+/−^-IP_3_R2^−/−^* and *IP_3_R1^−/−^-IP_3_R2^−/−^* placentas at E9.5. The area of the *IP_3_R1^−/−^-IP_3_R2^−/−^* labyrinth is significantly lower (*P<0.05, n = 3). Error bars indicate standard deviations. ev, embryonic vessel; mv, maternal vessel.
(5.68 MB TIF)

Figure S4 The percentages of cells with nuclear translocation of NFATc1 in the *IP_3_R1^−/−^-IP_3_R2^−/−^* hearts (white bars) are significantly lower than those in the *IP_3_R1^+/−^-IP_3_R2^−/−^* hearts (black bars) at E9.5 to E10.0 (*P<0.01, n = 3). Error bars indicate standard deviations.
(2.97 MB TIF)

Video S1 Atrioventricular regurgitation in the zebrafish hearts treated with DMSO (top), calcineurin inhibitor, cyclosporine A, (middle) and IP_3_R inhibitor, 2APB, (bottom).
(0.32 MB MOV)
