# Supplementary material for: Gene Knock-Outs of Inositol 1,4,5-Trisphosphate Receptors Types 1 and 2 Result in Perturbation of Cardiogenesis
Source: PLoS One. 2010 Sep 1;5(9):e12500. doi: 10.1371/journal.pone.0012500 (PMC2931702; doi:10.1371/journal.pone.0012500)
Supplement: Materials and Methods S1 — (0.03 MB DOC) [file pone.0012500.s001.doc]

**Supporting Information**

**Materials and Methods S1**

**Transmission electron microscopy.**

For transmission electron microscopy (TEM) of embryonic hearts, embryos were initially fixed overnight in HEPES buffer (pH 7.4) that contained 2% glutaraldehyde and 2% paraformaldehyde. Ultrathin sections were double-stained with uranyl acetate and lead citrate, and observed under the JEOL-1230 transmission electron microscope.
