# Supplementary material for: Gene Knock-Outs of Inositol 1,4,5-Trisphosphate Receptors Types 1 and 2 Result in Perturbation of Cardiogenesis
Source: PLoS One. 2010 Sep 1;5(9):e12500. doi: 10.1371/journal.pone.0012500 (PMC2931702; doi:10.1371/journal.pone.0012500)
Supplement: Table S1 — Genotype Distributions of Embryos from IP3R1+/−-IP3R2−/− Intercrosses. (0.03 MB DOC) [file pone.0012500.s002.doc]

Table S1.

Genotype Distributions of Embryos from *IP_3_R1^+/-^-IP_3_R2^-/-^* Intercrosses

| Embryonic day | N | *1^+/+^2^-/-^* (%) | *1^+/-^2^-/-^* (%) | *1^-/-^2^-/-^* (%) |
| --- | --- | --- | --- | --- |
| E9.0 | 23 | 35 | 43 | 22 |
| E9.5 | 205 | 31 | 53 | 16 |
| E10.0 | 35 | 26 | 51 | 23 |
| E10.5 | 121 | 20 | 52 | 28 |
| E11.5 | 16 | 44 | 56 | 0 |
