# Supplementary figures and images for: Gene Knock-Outs of Inositol 1,4,5-Trisphosphate Receptors Types 1 and 2 Result in Perturbation of Cardiogenesis
Source: PLoS One. 2010 Sep 1;5(9):e12500. doi: 10.1371/journal.pone.0012500 (PMC2931702; doi:10.1371/journal.pone.0012500)

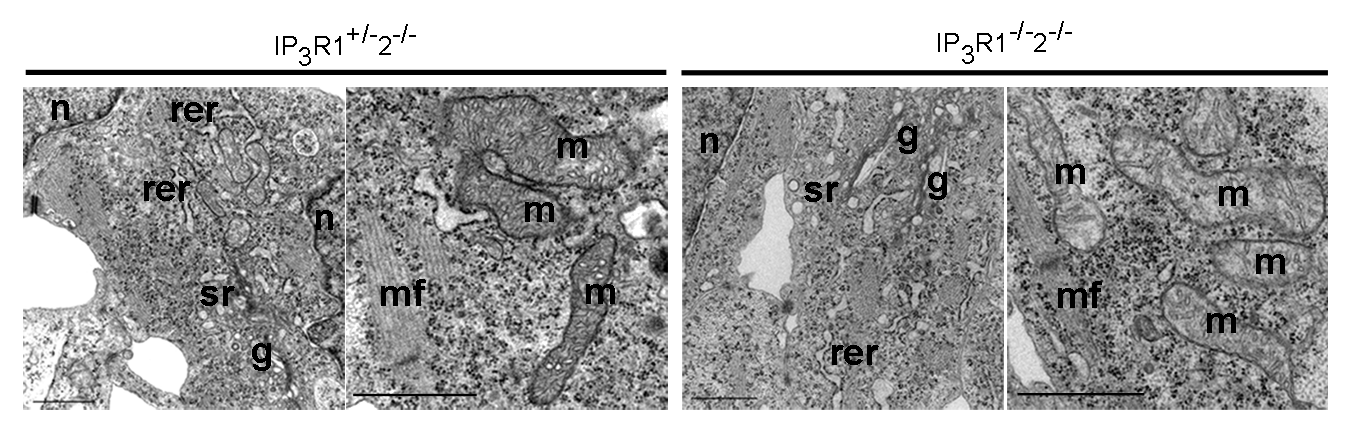

Supplement: Figure S1 — The ultrastructures of the subcellular organelles of the IP3R1−/−-IP3R2−/− mice are comparable with those of the IP3R1+/−-IP3R2−/− mice. Scale bars, 1 µm. g, golgi; m, mitochondrion; mf, myofilament; n, nucleus; rer, rough endoplasmic reticulum; sr, sarcoplasmic reticulum. (1.74 MB TIF) [file pone.0012500.s003.tif]

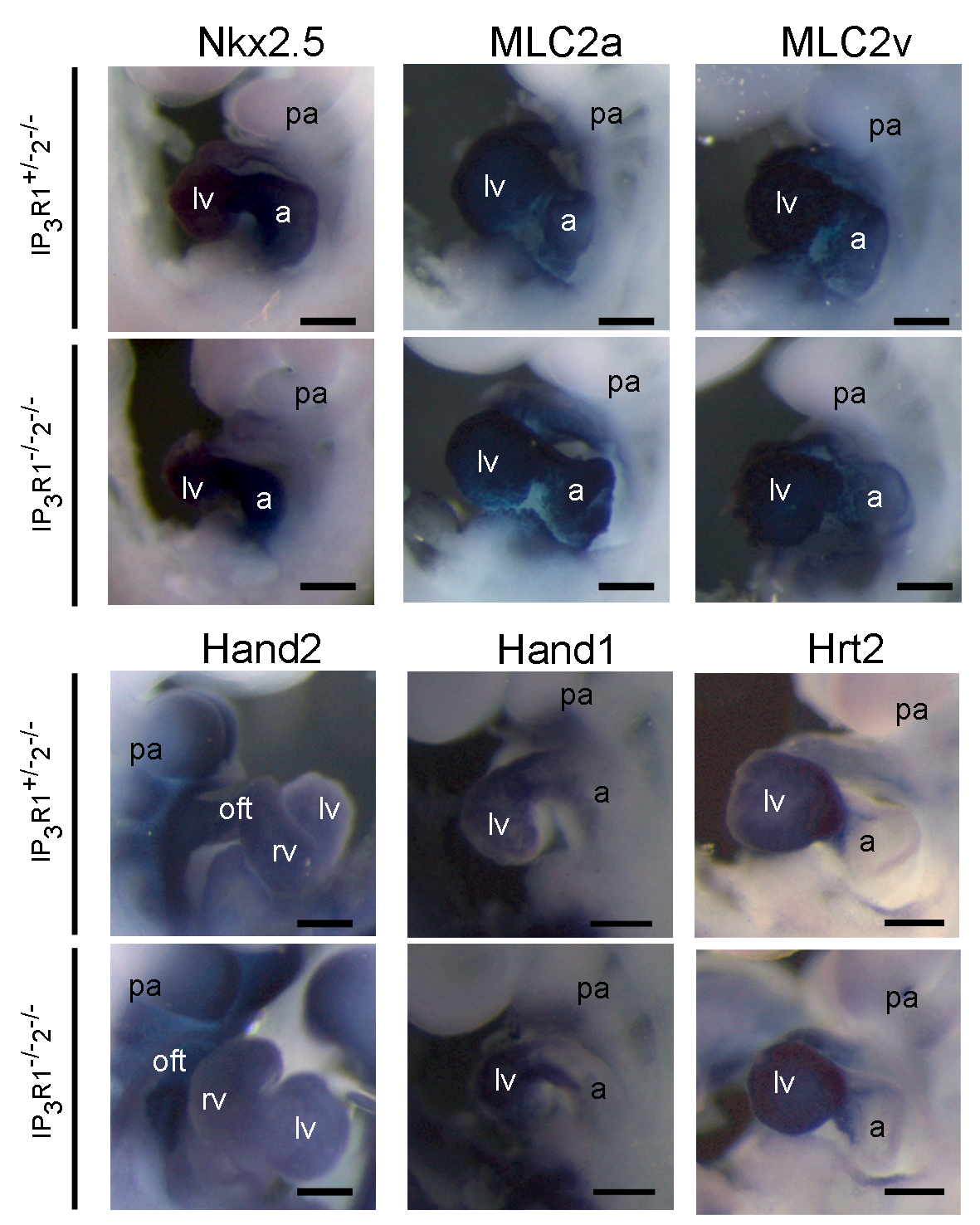

Supplement: Figure S2 — Expression of site-specific markers in embryonic hearts. Whole-mount in situ hybridization images of the left- or right-side of the hearts of E9.5 IP3R1+/−-IP3R2−/- (upper panels) and IP3R1−/−-IP3R2−/− (lower panels) embryos. The expression patterns of Nkx2.5, MLC2a, and MLC2v (earliest markers of the embryonic heart), and of Hand2, Hand1 and Hrt2 (markers of the right, left, and both ventricles, respectively) are shown. Scale bars, 0.2 mm. a, atrium; lv, left ventricle; oft, outflow tract; pa, pharyngeal arch; rv, right ventricle. (5.26 MB TIF) [file pone.0012500.s004.tif]

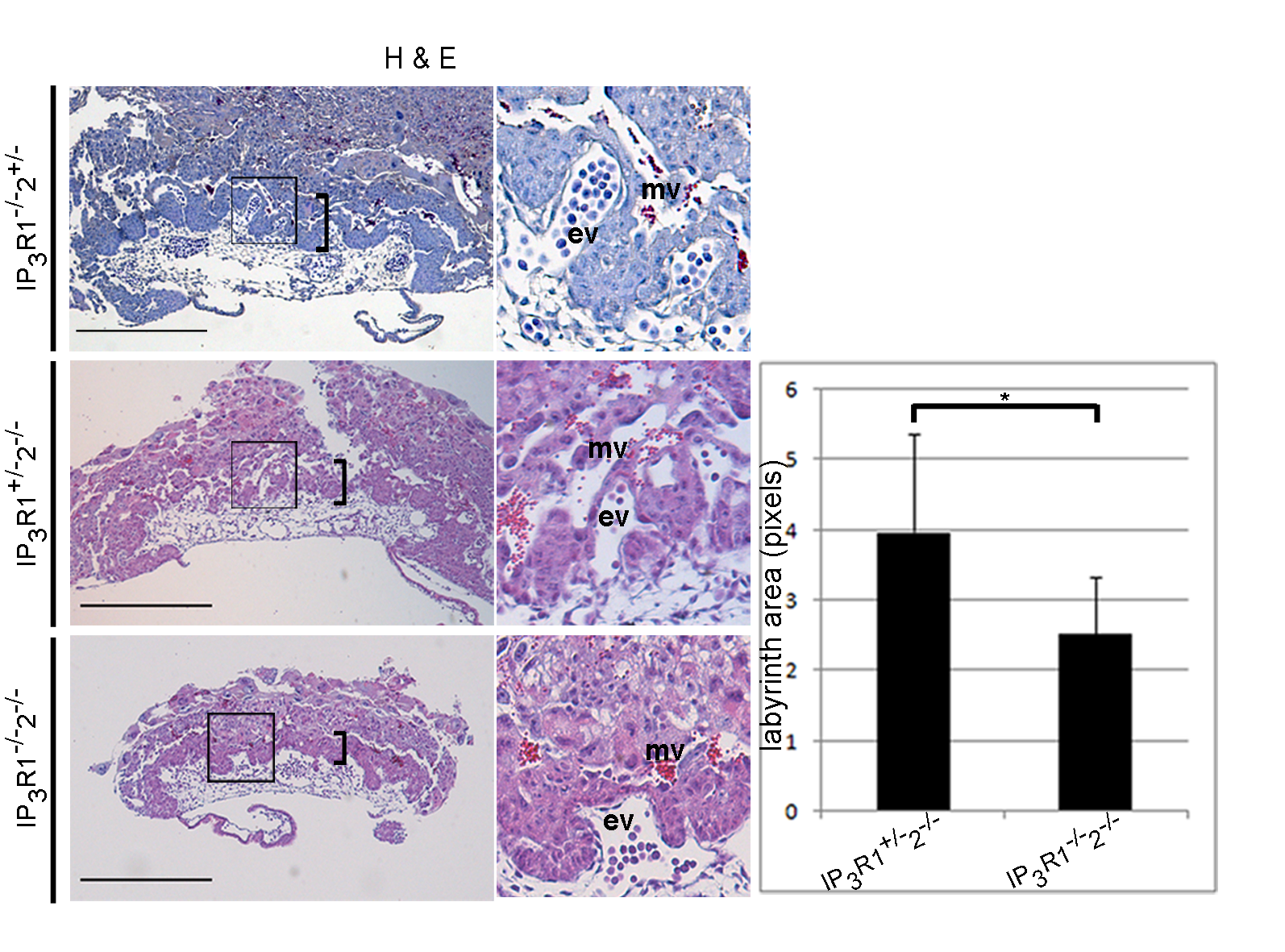

Supplement: Figure S3 — Cross-sections of E9.5 placentas from IP3R1−/−-IP3R2+/− (upper panels), IP3R1+/−-IP3R2−/− (middle panels) and IP3R1−/−-IP3R2−/− (lower panels) mutant mice. The widths of the labyrinth area indicated in parentheses. Higher-magnification images of the boxed areas are shown in the right panels. Scale bars, 0.5 mm. The graph shows quantification of the labyrinth areas of the IP3R1+/−-IP3R2−/− and IP3R1−/−-IP3R2−/− placentas at E9.5. The area of the IP3R1−/−-IP3R2−/− labyrinth is significantly lower (*P<0.05, n = 3). Error bars indicate standard deviations. ev, embryonic vessel; mv, maternal vessel. (5.68 MB TIF) [file pone.0012500.s005.tif]

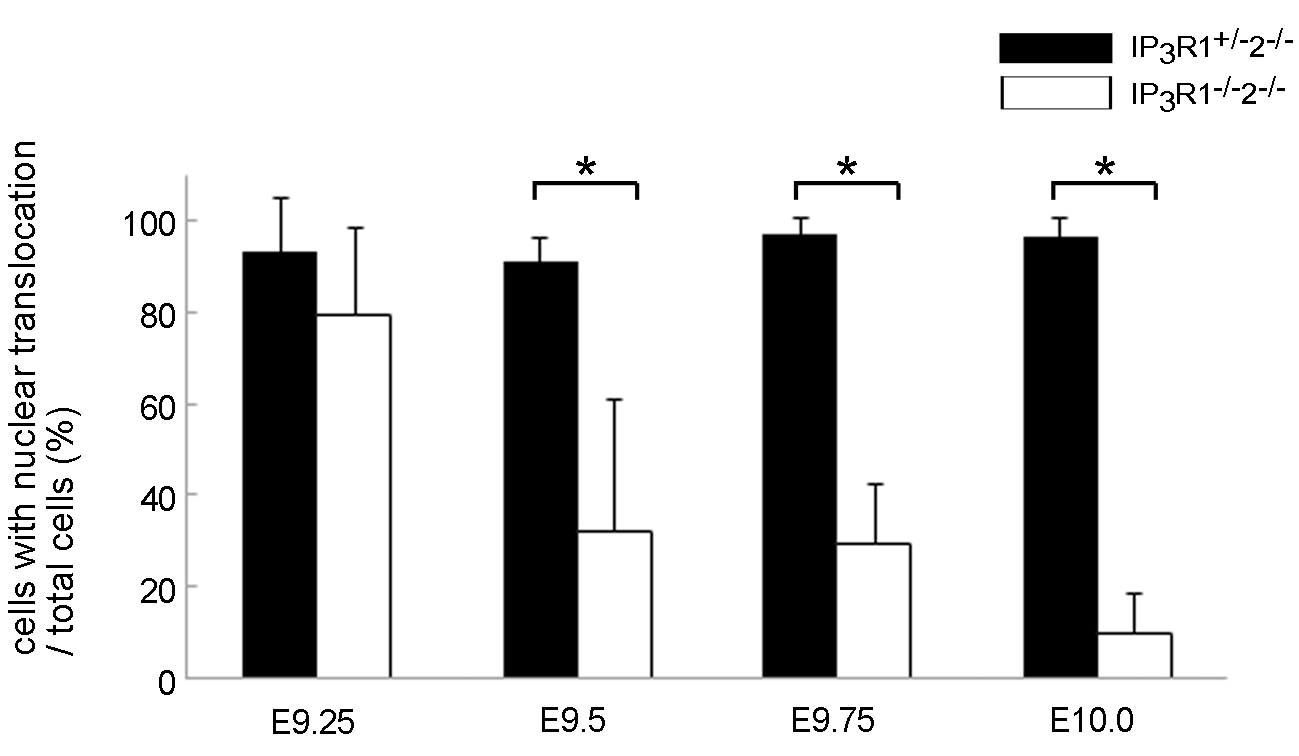

Supplement: Figure S4 — The percentages of cells with nuclear translocation of NFATc1 in the IP3R1−/−-IP3R2−/− hearts (white bars) are significantly lower than those in the IP3R1+/−-IP3R2−/− hearts (black bars) at E9.5 to E10.0 (*P<0.01, n = 3). Error bars indicate standard deviations. (2.97 MB TIF) [file pone.0012500.s006.tif]
